# Prediction of classical versus non classical papillary thyroid carcinoma subtypes from cytology of nodules classified according to TIRADS

**DOI:** 10.1007/s12020-023-03604-3

**Published:** 2023-11-25

**Authors:** Lorenzo Scappaticcio, Pierpaolo Trimboli, Giuseppe Bellastella, Pamela Ferrazzano, Eduardo Clery, Immacolata Cozzolino, Marco Montella, Morena Fasano, Mario Pirozzi, Sonia Ferrandes, Giovanni Docimo, Fortunato Ciardiello, Renato Franco, Katherine Esposito

**Affiliations:** 1 Unit of Endocrinology and Metabolic Diseases, AOU University of Campania “Luigi Vanvitelli”, Naples, Italy; 2 Department of Advanced Medical and Surgical Sciences, University of Campania “Luigi Vanvitelli”, Naples, Italy; 3 Clinic of Endocrinology and Diabetology, Lugano and Mendrisio Regional Hospital, Ente Ospedaliero Cantonale, Bellinzona, Switzerland; 4 Faculty of Biomedical Sciences, Università della Svizzera Italiana, Lugano, Switzerland; 5 Pathology Unit, Department of Mental and Physical Health and Preventive Medicine, University of Campania “Luigi Vanvitelli”, Naples, Italy; 6 Oncology Unit, Department of Precision Medicine, Università degli Studi della Campania “Luigi Vanvitelli”, Naples, Italy

**Keywords:** Thyroid nodules, PTC subtypes, TI-RADS

## Abstract

**Purpose:**

Our purposes were: 1) to estimate the prediction performance (PP) of cytology in identifying papillary thyroid carcinoma (PTC) subtypes; 2) to explore how the PTC subtypes distribute among the American College of Radiology (ACR) Thyroid Imaging Reporting and Data System (TI-RADS) categories.

**Methods:**

Nodules were included if both the histology with the PTC subtype report and the cytology report with the possible PTC subtype were available. The PP was calculated by making the proportion of True positives/False positives+false negatives.

**Results:**

309 cytologically “suspicious for malignancy” and “malignant” thyroid nodules with PTC histology were evaluated. ACR TI-RADS categorization for classical PTC was significantly different from non-classical PTC (*p-*value 0.02). For the whole cohort the PP of cytologically classical cases was 0.74, while that of cytologically non classical cases was 0.41. ACR TI-RADS categorization was not significantly different for aggressive vs non-aggressive PTC subtypes (*p-*value 0.1). When considering only aggressive or non-aggressive PTC subtypes, the PP of cytologically classical cases was respectively 0.86 and 0.87, while that of cytologically non classical cases was respectively 0.27 and 0.22. The PP of cytologically classical cases was 0.73 and 0.79, respectively for macroPTCs and microPTCs, while that of cytologically non classical cases was 0.55 and 0.33, respectively for macroPTCs and microPTCs.

**Conclusion:**

Cytology examination reliably performed in predicting classical PTC versus non classical PTC subtypes. ACR TI-RADS categorization was significantly different among classical PTC versus non classical PTC subtypes.

## Introduction

Papillary thyroid carcinoma (PTC) is not a unique entity as it encompasses several histological subtypes with heterogeneous biological behaviors [1–3].

According to the recent literature, aggressive subtypes of PTC are responsible for the majority of recurrences, increased morbidity, and shortened disease-free survival, suggesting that treatment should be tailored to specific histologic subtypes [3–5]. Thyroid lobectomy alone may be sufficient as well as the initial treatment for low risk PTC (i.e., microPTC, PTC > 1 cm and <4 cm) [1]. Moreover, for the treatment of the aggressive subtypes, the American Thyroid Association (ATA) recommends lobectomy (LT) for small unifocal intrathyroidal tumors, total thyroidectomy with therapeutic neck dissection if nodes are involved, or prophylactic central neck dissection for T3 and T4 tumors [1]. In 2015, the ATA guidelines [1] adopted active surveillance (AS) as an alternative to immediate surgery in select patients with low-risk PTC. At this point, as the rate of AS is increasing, preventing overdiagnosis and overtreatment of very low-risk PTCs is one of the most important issues, while predicting the aggressive subtype of PTC is similarly important and is likely a necessary task [6]. Moreover, thermal ablation techniques might be a promising feasible alternative to lobectomy in the treatment of selected low-risk microPTCs [7]. At present for LT or AS or thermal ablation therapies, candidate primary tumors should demonstrate a cytologic confirmation of papillary thyroid cancer without aggressive subtype [1, 7, 8].

US-based risk stratification systems (RSSs), often referred as Thyroid Imaging Reporting and Data Systems (TIRADSs), mainly apply to PTC, and not to other thyroid cancers [9–12]. Fine-needle aspiration cytology (FNAC) has been widely used as the most effective preoperative evaluation tool to detect PTC [13, 14]. The third edition of The Bethesda system for reporting thyroid cytopathology (TBSRTC) even included a detailed definition with particular criteria for some of these PTC subtypes but did not recommend formulating the diagnosis of a specific PTC subtype by cytology [15]. This is because the diagnosis of PTC subtypes by preoperative FNAC may not be easy or reliable even for a skilled cytopathologist [13–15]. Similarly, preoperative diagnosis of a PTC subtype using nUS is challenging, and the US characteristics and difference for various subtypes of PTC are still unclear [16]. However, US features at the time of diagnosis must be explored since they can serve as a useful tool for predicting biological behavior in PTC [17].

Subtyping PTC through cytology and US is clinically relevant as well as exploring other main characteristics of PTC such as size, lymph node metastasis, extrathyroidal extension, multifocality, personal/family history of PTC [5, 13, 16]. Indeed, aggressive histology, like tall cell, hobnail subtype, columnar cell, solid subtype, confers intermediate risk according to the current ATA guidelines [1].

Therefore, it is desirable to preoperatively identify or at least give some clues of the different subtypes of PTC [13, 18]. Yet, the preoperative identification of small classical PTCs would facilitate more conservative management (i.e., LT, AS, thermal ablation).

To the best of our knowledge, few studies evaluated the ability of preoperative FNAC to identify PTC subtypes [19–22]. Similarly, only emerging studies describing the US image characteristics of PTC subtypes have been published to date [16, 23–27].

Through the current study we want to explore the value of the FNAC report of classical versus (vs) non-classical PTC subtype relative to suspicious and malignant thyroid nodules classified according to TIRADS. Our purposes were: 1) to estimate the concordance between cytology and histology reports for classical vs non classical PTC subtypes; 2) to estimate the prediction performance of cytology in identifying classical vs non classical PTC subtypes; 3) to explore how the PTC subtypes distribute among the American College of Radiology (ACR) TIRADS categories.

## Methods

### Study design and patients

In the current study the Standards for Reporting Diagnostic Accuracy (STARD) statement was followed [28]. A retrospective analysis of consecutive FNAC results from adult patients with cytologically “suspicious for malignancy” and “malignant” thyroid nodules was carried out in our Academic referral center (University Hospital “L. Vanvitelli” - Naples, Italy) from January 2016 to December 2022. Nodules could be included whether: a) the matched histology after surgery with PTC subtype result was available; b) the FNAC result with the possible PTC subtype (i.e., classical vs non classical PTC) was available; c) the ACR TI-RADS categorization was applied separately from at least two clear B-Mode US images (i.e., transverse and longitudinal images). Nodules were excluded whether: a) they were associated with benign histology; b) they were associated with non-PTC malignant histology [i.e., follicular thyroid carcinoma, medullary thyroid carcinoma; poorly differentiated and anaplastic thyroid carcinoma, thyroid lymphoma, non-invasive follicular thyroid neoplasm with papillary-like nuclear features (NIFTP); cribriform morular thyroid carcinoma]; c) unavailability of well-preserved and adequate cytological samples.

The Ethics Committee of University Hospital “L. Vanvitelli” of Naples (Italy) approved the study protocol and all patients gave written informed consent.

### Thyroid ultrasonography

At our center US images were obtained by an ultrasound device (MyLab^™^Six, Esaote) with a 7–14 MHz wide band linear transducer. The color gain was adjusted so that artifacts were prevented. The examination of ultrasonographic features of thyroid nodules, along with thyroid vascularity and volume, were systematically conducted for patients presenting for thyroid assessment.

When reviewing the US images on digital format, two endocrinologists (G.B. and L.S. with 22 and 9 years of clinical experience, respectively, in performing and evaluating thyroid US) assessed the thyroid nodules by using the criteria of ACR TI-RADS [29], being unaware of nodule’s cytopathology and histopathology, of laboratory and imaging results. In case of disagreement on US categorization (i.e., TR1, TR2, TR3, TR4, TR5) [29] a consensus with the help of a third senior reviewer (P.T.) (also unaware of pathology or any other patient data) was reached.

### Thyroid nodule pathology

At our center US-guided FNAC was routinely performed by using a 23-gauge needle using a conventional method, and at least two needle passes were performed for each nodule. In all cases, direct air-dried smears were made after the FNAC procedure, then stained by using the May-Gruenwald-Giemsa (MGG) method. All the available slides from each case were reviewed. Indication to perform FNAC (also for nodules < 10 mm) was made by the endocrinologist according to US features, laboratory, other imaging (i.e., scintigraphy if performed), individual risk of malignancy, and patient/family preference.

At our center the cytologic diagnoses were reported according to the five subcategories of the revised Italian Consensus for the Classification and Reporting of Thyroid Cytology (ICCRTC) [30]. Moreover, for cytologically suspicious and malignant thyroid nodules a possible PTC subtype [i.e., cytologically classical PTC (cCLASS PTC) or cytologically non classical PTC (cNONCLASS PTC)] was routinely reported as an addendum to the final cytology report, using criteria previously identified as helpful for cytologic diagnosis of PTC subtype [5, 13]. However, cytologist could only report the suspicious of a classical vs non classical PTC without defining the final histotype [5, 13, 30].

All cytology specimens were reviewed by two thyroid cytopathologists (I.C. and M.M., with 20 and 10 years of clinical experience in thyroid cytopathology). At cytology in case of disagreement on PTC subtype (i.e., cCLASS PTC or cNONCLASS PTC), a consensus with the help of a third senior reviewer (R.F.) (also unaware of pathology or any other patient data) was reached. At all times, the two pathologists (I.C., M.M.) were unaware of histopathologic diagnoses made by other pathologists of our center. When reviewing cytology specimens to define the possible PTC subtype, they also were unaware of demographics and clinical data, including US features of thyroid nodules.

The final pathology [i.e., histology of the thyroid nodule after surgery and PTC subtype, histologically classical PTC (hCLASS PTC) and histologically non classical PTC (hNONCLASS PTC)] was made according to the 2022 WHO Classification of Thyroid Neoplasms [31]. At our center in this time, excluding NIFTP the risk of malignancy (ROM) of cytologically suspicious nodules was 81%, while including NIFTP among thyroid malignant entities this was 89.8%. At our center in this time, the ROM of cytologically malignant nodules was 100%. According to the 2022 WHO Classification [31], we split the non-classical PTC subtypes in aggressive [i.e., tall-cell (tc), solid (s), diffuse sclerosing (ds)] and non-aggressive [i.e., follicular variant (fv), oncocytic (o)]. Yet, Warthin-like PTC was considered an oncocytic PTC and cribriform-morular thyroid carcinoma cases were excluded since they were no longer classifed as a subtype of PTC [31].

PTC subtypes at histology (hCLASS PTC vs hNONCLASS PTC) were the reference standard both for the calculation of the overall concordance with cytology and the prediction performance of cytology reports. In the current study, relative to multifocal PTC cases consisting of one subtype we only contemplated the largest tumor focus submitted to FNAC. Moreover, in case of multifocal PTC cases consisting of more than one subtype or unifocal PTC cases made by more than one subtype, we only contemplated the largest and/or most aggressive PTC subtype submitted to FNAC. At the histopathology examination, 30% of the tumor focus was considered the cut-off value for the definition of an aggressive PTC subtype, except for solid PTC defined by ≥50% of solid growth [31].

### Statistical analysis

Continuous variables were described as mean and standard deviation. Categorical variables were presented as number (percentage). For the ACR TI-RADS distribution, we calculated the fashion (value at which the maximum frequency corresponded) and the *p*-values were calculated using the Chi-square test. In other cases, when required, observed level of significance (*p*-values), were calculated through the t-test. The overall concordance for PTC subtypes (i.e., classical vs non classical PTC) between cytology and histology was calculated using Cohen’s K test, where the kappa value (*k*) denotes the strength of agreement and is interpreted as follows: <0–0.2, poor; 0.21–0.4, fair; 0.4–0.6, moderate; 0.6–0,8, good; 0,8–1, very good. The prediction performance (PP) (i.e., the probability that from a cCLASS PTC case one would really get a hCLASS PTC case, and that from a cNONCLASS PTC case one would really get a hNONCLASS PTC case) was calculated by making the proportion of True positives/False positives+false negatives. The degree of error (DE) (i.e., the probability that from a cCLASS PTC case one would get a hNONCLASS PTC case and that from a cNONCLASS PTC case one would really get a hCLASS PTC case) was calculated as false negatives/false negatives + true positives. Statistical significance was defined as a *p-*value < 0.05. Statistical analysis was performed by MedCalc software version 9 (Mariakerke).

## Results

### Whole cohort characteristics

**Fig. 1:**
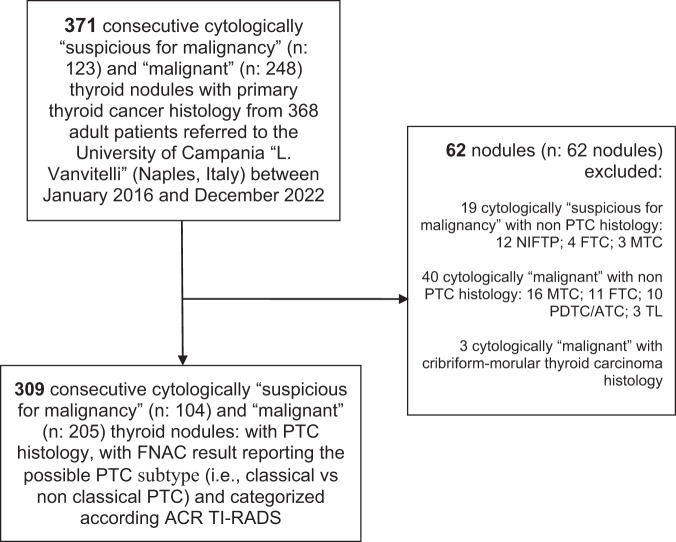
Flowchart of patients’ selection. PTC papillary thyroid carcinoma, FNAC fine needle aspiration cytology, ACR TI-RADS American College of Radiology Thyroid Imaging Reporting and Data Systems, NIFTP non-invasive follicular thyroid neoplasm with papillary-like nuclear features, FTC follicular thyroid carcinoma, MTC medullary thyroid carcinoma, PDTC/ATC poorly differentiated and anaplastic thyroid carcinoma, TL thyroid lymphoma, Cribriform-morular thyroid carcinoma is no longer classified as a subtype of PTC [31]

After applying our selection criteria, we finally included 309 cytologically “suspicious for malignancy” (n: 104) and “malignant” (n: 205) thyroid nodules with PTC histology from 309 patients (Fig. 1). Table 1 summarizes the main characteristics of the whole cohort.

**Table 1:** Main characteristics of the whole cohort (*n*: 309)

Characteristics	
Age at diagnosis, years (sd)	47.2 ± 14.9
Females/Males, *n* (ratio)	217/92 (2.4)
Maximal dimension, mm (sd)	14 ± 7.8 mm
• ≥10 mm, *n* (%)	202 (65.4)
• <10 mm, *n* (%)	107 (34.6)
ACR TI-RADS,	
• TR5, *n* (%)	171 (55.4)
• TR4, *n* (%)	128 (41.4)
• TR3, *n* (%)	10 (3.2)
Cytology,	
• cCLASS PTC, *n* (%)	259 (83.8)
• cNONCLASS PTC, *n* (%)	50 (16.2)
Histology,	
• hCLASS PTC, *n* (%)	222 (71.8)
• hNONCLASS PTC, *n* (%)	87 (28.2)
fv, *n* (%)	41 (47.1)
o, *n* (%)	24 (27.6)
tc, *n* (%)	14 (16.1)
s, *n* (%)	4 (4.6)
ds, *n* (%)	4 (4.6)

*sd* standard deviation; mm, millimeter, *ACR TI-RADS* American College of Radiology Thyroid Imaging Reporting and Data Systems, *PTC* papillary thyroid cancer, *cCLASS* cytologically classical PTC, *cNONCLASS* cytologically non classical, *hCLASS* histologically classical, *hNONCLASS* histologically non classical, *fv* follicular variant, *o* oncocytic, *tc* tall-cell, *s* solid, *ds* diffuse sclerosing

### Concordance and prediction performance of the whole cohort

Table 2 shows dimension and ACR TI-RADS categorization of histologically classical and non classical PTC, the concordance between cytology and histology and the PP of cCLASS and cNONCLASS PTC cases in the whole cohort. Overall concordance between cytology and histology was poor (*k* = 0.11). The PP of cCLASS PTC cases was 0.74 (with DE 0.26), while the PP of cNONCLASS PTC cases was 0.41 (with DE 0.59).

**Table 2:** Dimension and ACR TI-RADS categorization of histologically classical vs non classical PTC (A), cytohistologic concordance and prediction performance (PP) of cCLASS and cNONCLASS PTC cases (B) in the whole cohort (*n*: 309)

A				
Characteristics		CLASS PTC (*n*: 222)	NONCLASS PTC (*n*: 87)	*p*-value
Maximal dimension, mm (sd)		17.1 ± 12.1	13.2 ± 7.1	0.11
ACR TI-RADS,				
• TR5, n (%)		113 (50.9)	58 (66.7)	
• TR4, *n* (%)		105 (47.3)	23 (26.4)	
• TR3, *n* (%)		4 (1.8)	6 (6.9)	
Mode		TR5	TR5	0.02

B				
*Histology*	*Cytology*			
	cCLASS PTC, *n* (%)	cNONCLASS PTC, *n* (%)	total, *n (%)*	
hCLASS PTC, *n* (%)	193 (62.4)	29 (9.4)	222(71.8%)	
hNONCLASS PTC, *n* (%)	67 (21.7)	20 (6.5)	87 (28.2)	
total, n (%)			309 (100%)	
*p-*value			0.03	
weighted k			0.11	
PP, (DE)	0.74 (0.26)	0.41 (0.59)		

*sd*, standard deviation, *mm* millimeter, *ACR TI-RADS* American College of Radiology Thyroid Imaging Reporting and Data Systems *PTC*, papillary thyroid carcinoma, *cCLASS*, cytologically classical, *cNONCLASS* cytologically non classical, *hCLASS* histologically classical, *hNONCLASS* histologically non-classical, *PP* prediction performance, *DE* degree of error

*κ* value of <0–0.2 indicates a poor cytohistologic concordance

### Concordance and prediction performance considering only aggressive or non aggressive PTC subtypes

Table 3 shows dimension and ACR TI-RADS categorization of aggressive vs non aggressive NONCLASS PTC subtypes, the concordance between cytology and histology and the PP of cCLASS and cNONCLASS PTC cases when considering only aggressive or non aggressive NONCLASS PTC subtypes.

**Table 3:** Dimension and ACR TI-RADS categorization of aggressive vs non aggressive NONCLASS PTC subtypes (A), cytohistologic concordance and prediction performance (PP) of cCLASS and cNONCLASS PTC cases (B) when considering only aggressive or non aggressive NONCLASS PTC subtypes

A				
*C*haracteristics		Aggressive PTC (*n*: 22)	Non aggressive PTC (*n*: 65)	*p*-value
Maximal dimension, mm (sd)		16.3 ± 9.9	18.2 ± 13.5	0.76
ACR TI-RADS,				
• TR5, *n* (%)		13 (59.1)	45 (69.2)	
• TR4, *n* (%)		9 (40.9)	14 (21.6)	
• TR3, *n* (%)			6 (9.2)	
Mode		TR5	TR5	0.1

B				
*Histology*	*Cytology*			
Aggressive	cCLASS PTC, *n* (%)	cNONCLASS PTC, *n (%)*	total*, n* (%)	
hCLASS PTC, *n* (%)	50 (62.5)	8 (10.0)	58 (72.5%)	
hNONCLASS PTC, *n* (%)	16 (20.0)	6 (7.5)	22 (27.5)	
total, *n* (%)			80 (100%)	
*p*-value			0.02	
weighted k			0.16	
PP, (DE)	0.86 (0.14)	0.27 (0.73)		

Histology	*Cytology*			
Non aggressive	cCLASS PTC, *n* (%)	cNONCLASS PTC, *n* (%)	total, *n* (%)	
hCLASS PTC, *n* (%)	143 (62.4)	21 (9.2)	164(71.6%)	
hNONCLASS PTC, *n* (%)	51 (22.3)	14 (6.1)	65 (28.4)	
total, *n* (%)			229 (100%)	
*p*-value			0.03	
weighted k			0.17	
PP, (DE)	0.87 (0.13)	0.22 (0.78)		

*sd* standard deviation, *mm* millimeter, *ACR TI-RADS* American College of Radiology Thyroid Imaging Reporting and Data Systems, *PTC* papillary thyroid carcinoma, *cCLASS* cytologically classical, *cNONCLASS* cytologically non classical, *hCLASS* histologically classical, *hNONCLASS* histologically non classical, *PP* prediction performance, *DE* degree of error

*κ* value of <0–0.2 indicates a poor cytohistologic concordance

Aggressive non classical PTC subtypes include: tall-cell (tc), solid (s), diffuse sclerosing (ds)

Non aggressive non classical PTC subtypes include: follicular variant (fv), oncocytic (o)

When considering only aggressive subtypes the overall concordance between cytology and histology was poor (*k* = 0.16). When considering only non aggressive subtypes the overall concordance between cytology and histology was poor (*k* = 0.17). When considering only aggressive subtypes the PP of cCLASS PTC cases was 0.86 (with DE 0.14), while the PP of cNONCLASS PTC cases was 0.27 (with DE 0.73). When considering only non aggressive subtypes the PP of cCLASS PTC cases was 0.87 (with DE 0.13), while the PP of cNONCLASS PTC cases was 0.22 (with DE 0.78).

### Concordance and prediction performance of PTCs ≥ 10 mm

Table 4 shows dimension and ACR TI-RADS categorization of classical vs non classical PTC ≥ 10 mm, the concordance between cytology and histology and the PP of cCLASS and cNONCLASS PTC cases when considering only PTCs ≥ 10 mm.

**Table 4:** Dimension and ACR TI-RADS categorization of classical vs non classical PTC ≥ 10 mm (A), cytohistologic concordance and prediction performance (PP) of cCLASS and cNONCLASS PTC cases (B) when considering only PTCs ≥10 mm (*n*: 202)

A				
Characteristics		CLASS PTC (*n:* 138)	NONCLASS PTC (*n:* 64)	*p-*value
Maximal dimension, mm (sd)		15.1 ± 4.1	17.2 ± 6.3	0.34
ACR TI-RADS,				
•TR5, *n* (%)		69 (50.0)	48 (75.0)	
•TR4, *n* (%)		66 (47.8)	12 (18.7)	
•TR3, *n* (%)		3 (2.2)	4 (6.3)	
Mode		TR5	TR5	0.0003
**B**				
*Histology*	*Cytology*			
	cCLASS PTC, *n* (%)	cNONCLASS PTC, *n* (%)	*total, n (%)*	
hCLASS PTC, *n* (%)	122 (60.4)	16 (7.9)	138(68.3%)	
hNONCLASS PTC, *n* (%)	44 (21.8)	20 (9.9)	64 (31.7)	
total, *n (%)*			202 (100%)	
*p-*value			0.01	
weighted *k*			0.22	
*PP, (DE)*	0.73 (0.27)	0.55 (0.45)		

*sd* standard deviation, *mm* millimeter, *ACR TI-RADS* American College of Radiology Thyroid Imaging Reporting and Data Systems *PTC* papillary thyroid carcinoma, *cCLASS* cytologically classical, *cNONCLASS* cytologically non classical, *hCLASS* histologically classical, *hNONCLASS* histologically non classical, *PP* prediction performance, *DE* degree of error

*κ* value of 0.21–0.4 indicates a fair cytohistologic concordance

For PTC cases with md ≥10 mm (i.e., macroPTCs) the overall concordance between cytology and histology was fair (*k* = 0.22). For macroPTCs the PP of cCLASS PTC cases was 0.73 (with DE 0.27), while the PP of cNONCLASS PTC cases was 0.55 (with DE 0.45).

### Concordance and prediction performance of PTCs < 10 mm

Table 5 shows dimension and ACR TI-RADS categorization of classical vs non classical PTC, the concordance between cytology and histology and the PP of cCLASS and cNONCLASS PTC cases when considering only PTCs < 10 mm.

**Table 5:** Dimension and ACR TI-RADS categorization of classical vs non classical PTC < 10 mm (A), cytohistologic concordance and prediction performance (PP) of cCLASS and cNONCLASS PTC cases (B) when considering only PTCs <10 mm (*n*: 107)

A				
Characteristics		CLASS PTC (*n*: 84)	NONCLASS PTC (*n*: 23)	*p-*value
Maximal dimension, mm (sd)		7.0 ± 1.3	8.1 ± 1.8	0.5
ACR TI-RADS,				
• TR5, *n* (%)		44 (52.4)	10 (43.5)	
• TR4, *n* (%)		39 (46.4)	11 (47.8)	
• TR3, *n* (%)		1 (1.2)	2 (8.7)	
Mode		TR5	TR4	0.1

B				
*Histology*	*Cytology*			
	cCLASS PTC, *n* (%)	cNONCLASS PTC, *n* (%)	total*, n (%)*	
hCLASS PTC, *n* (%)	72 (67.3)	12 (11.2)	84(78.5%)	
hNONCLASS PTC, *n* (%)	17 (15.9)	6 (5.6)	23 (21.5)	
total, *n (%)*			107 (100%)	
*p-*value			0.04	
*weighted k*			0.1	
*PP, (DE)*	0.79 (0.21)	0.33 (0.67)		

*sd* standard deviation, *mm* millimeter, *ACR TI-RADS* American College of Radiology Thyroid Imaging Reporting and Data Systems, *PTC* papillary thyroid carcinoma, *cCLASS* cytologically classical, *cNONCLASS*, cytologically non classical, *hCLASS* histologically classical, *hNONCLASS* histologically non classical, *PP* prediction performance, *DE* degree of error

*κ* value of <0–0.2 indicates a poor cytohistologic concordance

For PTC cases with md < 10 mm (microPTCs) the overall concordance between cytology and histology was poor (*k* = 0.1). For microPTCs the PP of cCLASS PTC cases was 0.79 (with DE 0.21), while the PP of cNONCLASS PTC cases was 0.33 (with DE 0.67).

Table 6 summarizes the concordance between cytology and histology and the PP of cCLASS and cNONCLASS PTC cases in the different cohorts (i.e., whole cohort, considering only aggressive or non aggressive subtypes, PTCs ≥ 10 mm, PTCs < 10 mm).

**Table 6:** Cytohistologic concordance and prediction performance (PP) of cCLASS and cNONCLASS PTC cases in the different cohorts (i.e., whole cohort, considering only aggressive or non aggressive subtypes, PTCs ≥ 10 mm, PTCs < 10 mm)

	Whole cohort	Aggressive	Non aggressive	PTCs ≥ 10 mm	PTCs < 10 mm
*weighted k*	0.11	0.16	0.17	0.22	0.1
*PP, (DE) cCLASS*	0.74 (0.26)	0.86 (0.14)	0.87 (0.13)	0.73 (0.27)	0.79 (0.21)
*PP, (DE) cNONCLASS*	0.41 (0.59)	0.27 (0.73)	0.22 (0.78)	0.55 (0.45)	0.33 (0.67)

*PTC* papillary thyroid carcinoma, *cCLASS* cytologically classical, *cNONCLASS* cytologically non classical, *PP* prediction performance, *DE* degree of error

*κ* value of <0–0.2 indicates a poor cytohistologic concordance

*κ* value of 0.21–0.4 indicates a fair cytohistologic concordance

## Discussion

The performance of cytology and ultrasound in identifying different morphologic subtypes of PCT are promising [13, 16]. Because of the significant implications for patient management, distinction of CLASS PTC from NONCLASS PTC is desirable at the time of preoperative FNAC and ultrasound [1, 7]. Moreover, to triage patients with small CLASSPTC to more conservative clinical management, cytopathologists and ultrasonographers need to be able to distinguish CLASS PTC from NONCLASS PTC [1, 7].

To determine if this distinction can be made in a reliable manner, we retrospectively classified a large series of PTC and its subtypes in the 6-year range, cytologically diagnosed as “suspicious for malignancy” or “malignant” using cytologic criteria previously identified as helpful [5, 13] and ACR TI-RADS categorization [29]. This cohort finally included 309 nodules with PTC histology having a mean md of 14 ± 7.8 mm, about two-thirds with md ≥ 10 mm. According to current evidence [17, 32], our nodules mainly distributed in high risk TI-RADS categories (i.e., over 95% were TR5 and TR4, and less than 5% were TR3). Overall, we found that the cytology exam overestimated the percentage of classical PTC cases while it underestimated the percentage of non-classical PTC cases: indeed, histology of PTC subtypes revealed about 10% less of classical PTC and about 10% more of non-classical PTC cases compared to cytology. Thus, histology confirmed classical PTC in about 70% of the whole cohort, with the remaining cases corresponding to non-classical subtypes of PTC (i.e., almost 50% fv, less than 30% o, about 15% tc, about 5% both s and ds). In this way, our final cohort correctly depicted the actual prevalence of PTC subtypes, as conventional PTC typically covers the 70% of PTCs, and fv and o are the main two non classical PTC subtypes [1, 5, 18].

Classical and non-classical PTCs were similar as regards mean md, while they significantly differently distributed according to ACR TI-RADS: indeed, although the highest risk category (TR5) was the mode for both, more intermediate (TR3) and highest risk nodules were found in the group of non-classical PTCs. This finding was likely due to the evidence that, compared to classical PTC, fv and o PTC more frequently could appear as solid, isoechoic and non-suspicious nodules at nUS, while aggressive PTC subtypes (i.e., tc, s, ds) usually have more than three US high risk features [16, 24, 25, 27]. For example, as reported by Zhang et al. [24], compared to classical PTC, fv PTC more frequently fell into TIRADS 3 and 4 categories.

Starting from a cytology report of possible CLASSPTC vs NONCLASS PTC, the cytohistologic concordance was collectively poor as expressed by the *κ* value of 0.11. This result would mean that cytology was not reliable to predict the final histology represented by the classical vs non classical PTC subtype. However, this issue was significantly different depending on whether the initial cytology suspicion was represented by CLASSPTC or NONCLASS PTC. Indeed, we found that prediction performance of cytology suspicion for classical PTC (i.e., the probability that from a cCLASS PTC case one would really get a hCLASS PTC case) was more than 70%: in other words, the cytology suspicion of classical PTC would be confirmed by histology in three out of four cases. Conversely, we found that prediction performance of cytology suspicion for non-classical PTC (i.e., the probability that from a cNONCLASS PTC case one would really get a hNONCLASS PTC case) was less than 50%: in other words, the cytology suspicion of non-classical PTC would be confirmed by histology in less than 2 out of four cases. High prediction performance was observed in the diagnosis of CLASSPTC due to supposed easier recognition of its classic cytologic features on FNAC compared to NONCLASSPTCs [13, 21]. However, poor literature to date evaluated the ability of preoperative FNAC to identify PTC subtypes [19–22]. The results of these studies collectively were in line with our findings: specifically, in the study by Nair et al. [20] of the 91 cases of CLASSPCT, 65 cases were correctly typed as CLASSPCT by cytology, while only 50% (3 out of 6) of cases diagnosed as fv PTC on FNAC were confirmed on histology [20]; in the study by Gupta et al. [21] subclassification was correct in 87 of 96 (90.6%) cases of classic papillary carcinoma and in 25 of 43 (58.1%) of the other subtypes of PTC; yet, in the study by Cipolletta et al. [22] agreement was achieved in 40/63 cases of classic PTC (63.5%) while a heterogeneous agreement was found relative to non classic PTC subtypes. The main reasons for disagreement were as follows: (I) many PTCs were heterogeneous with more than one cell type and/or growth pattern, and the predominant pattern might not have been sampled by FNAC; (II) the cytomorphologic features among different PTC subtypes had significant overlap; the rarity of these aggressive subtypes makes it very difficult for cytopathologists to become familiar with their morphologic features [14].

In addition to the analyses in the whole cohort, we explored the same objectives in subgroups. First, we would explore ACR TI-RADS categorization for non-classical PTCs subdivided in aggressive (i.e., tc, s, ds) vs non-aggressive subtypes; then, we would calculate the PP when excluding from the whole cohort the aggressive or the non-aggressive non classical PTC subtypes to corroborate the PPs of the whole cohort. We found that aggressive PTC subtypes were not different from the non-aggressive subtypes regarding both dimension and ACR TI-RADS categorization. Compared to aggressive PTCs, although about 10% of non-aggressive PTCs also fell into the US intermediate risk category due to the not uncommon unsuspicious appearance [16, 24, 25, 27], TR5 was the mode in both groups. The unreliability of TIRADS for PTC subtypes was reported in the study by Baek et al. [25], where it was suggested that ultrasonographic features were not useful for distinguishing PTC subtypes. When we analyzed only non-aggressive or aggressive non classical PTC subtypes from the whole cohort, we obtained similar findings to that seen in the whole cohort: indeed, we found poor cytohistologic concordances and high PPs of cCLASS PTC (i.e., more than 80% in both scenarios), while low PPs of cNONCLASS PTC (i.e., about 25% in both scenarios). In other terms, the presence in the whole cohort of aggressive or non-aggressive non classical PTC subtypes tackled in isolation and one-by-one led to the same findings of the whole cohort, namely: a high ability of cCLASSPTC to predict hCLASSPTC (with a low error risk), compared to the inadequacy of cNONCLASS to predict hNONCLASS PTC.

Moreover, considering the relevance of separating PTC ≥ 10 mm from PTC < 10 mm from a therapeutic point of view, we decided to assess if the ability of cytology to correctly distinguish classical from non-classical PTC subtypes could vary depending on the size of the primary PTC. Similarly, we want to explore the ACR TI-RADS categorization of classical vs non classical PTC when considering only PTCs ≥ 10 mm or PTCs < 10 mm. For the cohort of PTCs ≥ 10 mm, classical PTC significantly differently distributed in US risk categories vs non-classical PTCs according to ACR TI-RADS: indeed, like the whole cohort, non-classical PTC variants more frequently fell into TR5 and TR3 categories. Although an overall fair cytohistologic concordance (with a *κ* value of 0.22), for PTCs ≥ 10 mm starting from a cytological suspicion of classical PTC were confirmed in about three out of four cases; conversely the risk of error in predicting a non-classical PTC subtype from a cytological suspicion of non-classical PTC was one in two cases. For the cohort of PTCs < 10 mm, classical PTC similarly distributed in US risk categories vs non classical PTCs according to ACR TI-RADS, and this could be due to the difficulty to detect the US high-risk features in small nodules or the similar morphological appearance when PTCs are < 10 mm. Although an overall poor cytohistologic concordance (with a *κ* value of 0.1), for PTCs < 10 mm starting from a cytological suspicion of classical PTC were confirmed in eight out of ten cases; conversely the risk of error in predicting a non classical PTC subtype from a cytological suspicion of non-classical PTC was about seven out of ten cases. Therefore, ACR TI-RADS could mainly help to distinguish classical vs non classical PTC when the primary PTC was ≥ 10 mm.

Yet, the preoperative cytological suspicion of classical PTC appeared to be reliable in predicting classical PTC after surgery in all the cohorts (i.e., whole cohort, when considering only aggressive or non-aggressive PTC subtypes, PTCs ≥ 10 mm, PTCs < 10 mm). Meanwhile, the preoperative cytological suspicion of non-classical PTC appeared to be unreliable in predicting non classical PTC after surgery in all the cohorts.

By integrating our US and cytohistologic findings for PTC, we could accurately predict some clinical scenarios of cytologically “suspicious for malignancy” and “malignant” thyroid nodules, with potential impact on therapeutical strategies: i.e., when we face US high-risk nodules along with cytology suspicion of classical PTC we could be quite confident that we will find classical PTC at histology; conversely, when we face US high-risk nodules along with cytology suspicion of non-classical PTC we could expect classical or non-classical PTC in similar proportions at histology.

### Limitations

There are limitations of our study that warrant some caution. First, there was an unavoidable selection bias because the data for all patients were retrospectively evaluated. Second, results referred to the Italian consensus for the classification and reporting of thyroid cytology and ACR TI-RADS categorization from a single academic center. Third, results regarding the PPs and ACR TI-RADS categorization referred only to classical vs non classical PTC subtypes, without reference on specific non classical PTC subtypes. However, this study carried out a reliable attempt to preoperatively distinguish classical vs non classical PTC for routine clinical practice. Fourth, the cytology reports were based on the conventional slide method and not on the liquid-based cytology, but the applied criteria were mainly studied for conventional smear methodology [13]. Fifth, a relatively high proportion of classical PTCs was included because of the low incidence of other PTC subtypes, and this could have affected our results. Sixth, molecular analysis was not taken into account, although it seems that sonographic features of PTC subtypes correlate with their molecular drivers [33]. Seventh, there was lack of information on staging, clinical aggressiveness and prognosis of PTCs.

### Strenghts

This was the largest study to date on the role of preoperative FNAC in identifying classical vs non classical PTCs stratified according to TIRADS. Moreover, this study was based on a heterogeneous distribution of PTC subtypes reflecting similar percentages of PTC subtypes reported in literature in patients with thyroid cancer [1, 5, 18]. Moreover, we did not just look at pathology data but we correlated them with ultrasonographic findings, as this is mandatory in daily clinical practice. Another strength was the blinded retrospective review by cytopathologists and ultrasonographists of the thyroid dataset. Yet, we made subgroup analyses considering only aggressive or non aggressive PTC subtypes and separating PTCs ≥ 10 mm and PTCs < 10 mm.

## Conclusion

The current study demonstrated that: cytology examination reliably performed in predicting classical PTC, compared to non classical PTC subtypes; and, ACR TI-RADS categorization was significantly different among classical PTC vs non classical PTC subtypes, mainly for PTCs ≥ 10 mm.

Basically, we carried out a feasible and appropriate attempt to predict classical PTC by cytology of “suspicious for malignancy” and “malignant” thyroid nodules classified according ACR TI-RADS (i.e., PTCs < 10 mm and PTC ≥ 10 mm). By contrast, this attempt was demonstrated to be unreliable for non classical PTCs (i.e., aggressive and non aggressive PTC subtypes).

In this respect, thyroidologists should attempt to diagnose and appropriately triage most patients with putative classical PTC for consideration of the best management. It was our hope that the findings of our study could be validated in other reference centers to promote the best initial choice of treatment of cytologically “suspicious for malignancy” and “malignant” thyroid nodules.

## Data Availability

Data are available on request due to local (academic) restrictions.
